# Changes in Ocular Biometry Following PreserFlo MicroShunt Implantation and Trabeculectomy: A Prospective Observational Study

**DOI:** 10.7759/cureus.56188

**Published:** 2024-03-14

**Authors:** Shunsuke Nakakura, Satomi Oogi, Etsuko Terao, Yuki Nagata, Yasuko Fujisawa, Saki Dote, Kanae Ueda

**Affiliations:** 1 Ophthalmology, Saneikai Tsukazaki Hospital, Himeji, JPN

**Keywords:** axial length, corneal curvature, visual acuity, central corneal thickness, ocular biometry, preserflo microshunt, trabeculectomy, glaucoma

## Abstract

Background

This study aimed to evaluate postoperative changes in ocular biometry following initial PreserFlo MicroShunt implantation and trabeculectomy.

Methodology

This prospective, observational study analyzed 27 cases of PreserFlo MicroShunt implantation and 29 cases of trabeculectomy performed by a single surgeon. Visual acuity, intraocular pressure, corneal curvature, central corneal thickness, anterior chamber depth, and axial length were assessed at baseline and postoperatively at one day, one week, two weeks, one month, two months, three months, and six months. Patients requiring additional surgery and those with missing data were excluded. Consecutive data were compared with the baseline values using multiple comparisons.

Results

In both groups, intraocular pressure was significantly decreased from baseline at all postoperative time points (all p < 0.01). Visual acuity decreased in both groups at one day and one week postoperatively. Corneal curvature remained unchanged in both groups throughout the six-month follow-up. Central corneal thickness increased at one day and one week postoperatively in the PreserFlo group, but not in the trabeculectomy group. Anterior chamber depth exhibited a significant decrease at one week postoperatively in both groups. Axial length significantly decreased postoperatively until three months in the PreserFlo group and at all postoperative time points in the trabeculectomy group.

Conclusions

Ocular biometry following PreserFlo and trabeculectomy had a similar tendency postoperatively.

## Introduction

Glaucoma is a chronic, progressive, degenerative disease of the optic nerve that produces characteristic visual field defects. Currently, it is the second leading cause of blindness globally [1]. Lowering intraocular pressure (IOP) remains the sole evidenced treatment approach, as a 1-mmHg increase in IOP confers a 10%-18% increased risk of glaucoma [2-4]. Conversely, a 1-mmHg decrease in IOP leads to an estimated 10% reduction in glaucoma progression [5]. Recently, minimally invasive glaucoma surgeries have gained popularity worldwide as alternatives to conventional trabeculectomy (TLE) [6-9].

Among the minimally invasive glaucoma surgeries, both XEN and PreserFlo (Santen Pharmaceutical Co., Osaka, Japan) are categorized as minimally invasive “bleb” devices given their mechanism of diverting aqueous humor to the subconjunctival space, similar to filtering surgeries. PreserFlo more strongly reduces IOP than XEN [10,11], although its outcomes remain inferior to those of TLE [12,13]. However, PreserFlo might cause fewer postoperative complications than TLE [13], require a less steep surgical learning curve [14,15], and bridge the gap between microinvasive glaucoma surgery and traditional filtering surgery [16]. There are limited data on detailed changes in visual acuity and ocular biometry following PreserFlo implantation [17,18], as data have only been reported for up to one week [18] and three months after surgery [17]. Therefore, the current study evaluated changes in visual acuity and ocular biometry following initial PreserFlo and TLE surgeries performed by a single consultant surgeon.

## Materials and methods

This prospective, observational study was approved by the Institutional Review Board of Saneikai Tsukazaki Hospital (IRB number: 221050), and the study protocol adhered to the principles of the Declaration of Helsinki. Patients were recruited from the Department of Ophthalmology at Saneikai Tsukazaki Hospital between January 2023 and May 2023. The initial cohort comprised all patients undergoing either PreserFlo or TLE surgery performed by a single surgeon (SN). The inclusion criteria were as follows: (1) undergoing either initial PreserFlo or TLE surgery; (2) capable of adhering to an approximately six-month postoperative regimen at our facility; (3) agreed to accurate ocular biometry measurements; and (4) had study eligibility for either eye, with only the right eye included for analysis. The exclusion criteria were as follows: (1) requiring additional glaucoma surgery in the operating theater (including bleb needling) to control IOP within six months; (2) undergoing simultaneous cataract extraction; (3) prior glaucoma surgery within three years; (4) missing any data within six months; and (5) experiencing loss of light perception within six months.

The initial cohort comprised 38 eyes undergoing initial PreserFlo implantation and 37 eyes undergoing initial TLE. In the PreserFlo group, seven eyes were excluded because they required open bleb revision surgery, one eye was excluded because of loss of light perception, and three eyes with missing data were excluded. In the TLE group, three eyes requiring open bleb revision and five eyes with missing data were excluded. The final cohort comprised 27 PreserFlo and 29 TLE cases. In the PreserFlo group, seven eyes were inserted in the temporal superior conjunctival field and 20 eyes were inserted in the temporal inferior conjunctival field. In TLE groups, all eyes were operated in the temporal superior conjunctival field. For the PreserFLo group, patient selection for positioning the PreserFLo device in the inferior area was based on either a history of prior filtering surgery in the temporal region or a relatively younger age. The optimal surgical procedure for each patient’s circumstances was determined by the surgeon.

Surgical techniques and postoperative management

For PreserFlo, the eye was retracted at the cornea using 7-0 silk sutures. A 7-mm limbal incision and a 2-mm radial conjunctival incision were made, sub-Tenon anesthesia was performed with 2% lidocaine with epinephrine, and hemostasis was performed. Spaces were created under both Tenon’s capsule and the conjunctiva for enhanced surgical visualization, followed by electrocautery for hemostasis. A neurosurgical pledget soaked in 0.04% mitomycin C (MMC) was placed below Tenon’s capsule and the conjunctiva for three minutes before irrigation with approximately 50 mL of balanced salt solution. A 1-mm corneal side port was made, and an incision was made 3.5 mm from the limbus, ensuring that the knife tip entered the anterior chamber. The PreserFlo device was inserted, and patency was confirmed by injecting a balanced salt solution through the side port and visualizing the flow from the device. The conjunctiva and Tenon’s capsule were closed with 8-0 Vicryl sutures overlying the PreserFlo plate. One month after surgery, residual conjunctival 8-0 Vicryl sutures were removed to reduce surgically induced astigmatism.

For TLE, a 7-0 silk corneal traction suture was placed to fixate the eye inferiorly. A 7-mm limbal conjunctival incision was made, and sub-Tenon anesthesia was administered using 2% lidocaine with epinephrine for intraoperative hemostasis. Spaces were created under Tenon’s capsule and the conjunctiva to enhance visualization. Cauterization achieved hemostasis, and a 2.5 × 2.5 mm rectangle scleral flap was created. A neurosurgical pledget soaked with 0.04% MMC was placed under Tenon’s capsule and the conjunctiva for three minutes before washing with approximately 50 mL of balanced salt solution. A posterior 2.0 × 2.0 mm scleral flap was fashioned and excised to form an intrascleral tunnel. After creating an anterior chamber entry with a V-lance and Kelly punch, peripheral iridectomy was performed. The scleral flap was sutured with approximately four or five interrupted 10-0 nylon sutures and tightened to achieve intraoperative IOP of 10-15 mmHg. The conjunctiva was sutured by 10-0 nylon using wing sutures and one horizontal block suture. Postoperative laser suturelysis was performed at one week targeting IOP of 11-12 mmHg and IOP of 8-9 mmHg at two weeks. One month after surgery, residual scleral flap sutures were done laser suturelysis to reduce surgically induced astigmatism

Postoperatively, both groups were treated with topical 1.5% levofloxacin and 0.1% betamethasone four times daily for approximately three months with a gradual taper. Both surgeries, postoperative care, and laser suture lysis procedures in TLE were performed by the same surgeon (SN) to minimize confounding from variations in surgical technique, bleb management strategies, skill, and other variables.

Ocular biometry measurements

Best-corrected visual acuity (BCVA) was assessed using a Landolt chart. Corneal curvature was defined as the mean of the horizontal (K1) and vertical (K2) corneal curvatures measured using an autorefractor keratometer (KR-800A; Topcon, Tokyo, Japan). Anterior chamber depth (distance from the corneal endothelium to the lens surface) and central corneal thickness were measured by three-dimensional corneal and anterior-segment optical coherence tomography (SS-1000 CASIA2; Tomey, Nagoya, Japan), and the mean of three measurements was adopted. Axial length was measured using an IOL Master 700 system (Carl Zeiss Meditec, Jena, Germany), and the mean of five measurements was adopted. After visual acuity and ocular biometry were assessed, IOP was evaluated via Goldmann applanation tonometry.

Statistical analyses

Statistical analyses were conducted using BellCurve for Excel (Version 4.05). Fisher’s exact test, the chi-squared test, and Welch’s t-test were used to compare patient background and postoperative complication frequencies. Changes in IOP, BCVA, corneal curvature, central corneal thickness, anterior chamber depth, and axial length following surgery were analyzed with a repeated-measures analysis of variance (ANOVA) and Bonferroni correction to compare the values before surgery (pre) and at each time point. For analyses, BCVA determined using the Landolt chart was converted to logarithm of the minimum angle of resolution (logMAR) units. Count fingers, hand motion, light perception, and no light perception were assigned logMAR values of 1.9, 2.3, 2.7, and 3.0, respectively [19]. Fixed-combination medications were considered as two medications, whereas oral acetazolamide was considered a single medication. A P-value <0.05 was considered statistically significant.

## Results

Table 1 comprehensively presents demographic information about 27 eyes from 27 patients in the PreserFlo group and 29 eyes from 29 patients in the TLE group. No statistically significant differences were identified in terms of sex, the treated eye, median age, previous glaucoma surgery, and the number of glaucoma medications between the PreserFlo and TLE cohorts (all p > 0.05). Notably, the PreserFlo group featured a higher proportion of patients with exfoliation glaucoma than the TLE group (p = 0.013, chi-squared test). The median age was relatively advanced in both groups (74 years vs. 67 years; p = 0.125, Welch’s t-test), whereas the incidence of intraocular lens usage (p = 0.006, Fisher’s exact test) and elevated preoperative IOP (p < 0.001, Welch’s t-test) was significantly higher in the PreserFlo group than in the TLE group. Furthermore, the HFA mean deviation was significantly worse in the TLE group. For preoperative ocular biometry, central corneal thickness and anterior chamber depth were different between the groups (all p < 0.005). However, BCVA, central curvature, and axial length were similar between the groups (p = 0.809 and 0.899, respectively).

**Table 1:** Patient demographics. P-values were calculated using Fisher’s exact test, the chi-squared test, or Welch’s t-test. Significant values were shown as bold. MD = mean deviation; SD = standard deviation; IOP = intraocular pressure; TLE = trabeculectomy, IOL = intraocular lens; BCVA = best-corrected visual acuity

	PreserFlo	TLE	P-value
Number at baseline	27	29	
Sex (female), %	9 (33)	13 (44)	0.272
Treated eye, right (%)	13 (48)	10 (34)	0.221
Median age (quantiles)	74 (58, 82)	67 (56, 75)	0.125
Lens status, IOL (%)	20 (74)	11 (37)	0.006
Glaucoma type			0.013
Primary open-angle glaucoma (n, %)	14 (52)	25 (86)	
Exfoliation glaucoma (n, %)	10 (37)	2 (7)	
Secondary glaucoma (n, %)	3 (11)	2 (7)	
Previous glaucoma surgery			
Selective laser trabeculoplasty (n, %)	4 (14)	1 (3)	0.154
Ab externo trabeculotomy (n, %)	2 (7)	2 (7)	0.666
Ab interno trabeculotomy (n, %)	3 (11)	0	0.105
TLE (n, %)	3 (11)	0	0.105
HFA (30-2 or 24-2 program) MD (mean, SD)	−10.2 (8.2)	−17.9 (8.0)	0.002
Baseline biometry (mean, SD)			
BCVA (logMAR)	0.17 (0.50)	0.04 (0.32)	0.266
Corneal curvature (mm)	7.63 (0.27)	7.75 (0.28)	0.809
Central corneal thickness (μm)	540 (37)	507 (37)	0.001
Anterior chamber depth (mm)	3.80 (0.64)	3.38 (0.74)	0.028
Axial length (mm)	25.0 (1.59)	25.0 (1.83)	0.899
Number of glaucoma medications at baseline, (mean, SD)	3.2 (0.8)	3.5 (0.9)	0.287
Preoperative IOP (mmHg), (mean, SD)	28.8 (8.4)	21.8 (5.5)	<0.001
IOP at 6 months (mmHg), (mean, SD)	14.1 (2.4)	11.6 (2.2)	<0.001

**Figure 1:**
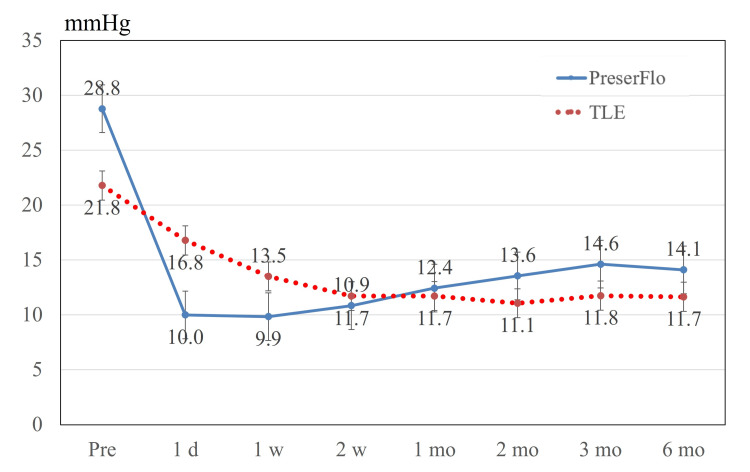
IOP during the follow-up period. IOP was significantly decreased at all time points (all p < 0.001, Dunnett’s test) in both groups after surgery. In the PreserFlo group, the lowest intraocular pressure was 9.9 mmHg one week after surgery. In the TLE group, the lowest IOP was 11.1 mmHg two months post-surgery. Values are shown as mean ± standard error of the mean. IOP = intraocular pressure; TLE = trabeculectomy

Figure 1 presents IOP changes after the PreserFlo and TLE procedures. IOP was significantly reduced at all time points after surgery in both groups (all p < 0.001, Dunnett’s test). Specifically, in the PreserFlo group, the baseline IOP was 28.8 mmHg, whereas it reached a nadir of 9.9 mmHg one week after surgery. In the TLE group, the baseline IOP was 21.8 mmHg, and the lowest recorded IOP of 11.1 mmHg was observed two months post-surgery. In the PreserFlo group, two patients resumed glaucoma medications (bimatoprost 0.03% and carteolol 2% hydrochloride/latanoprost 0.005% fixed-combination) at 94 and 169 days, postoperatively, respectively, whereas the remaining 25 patients remained medication-free at the six-month follow-up. Conversely, no patients in the TLE group required glaucoma medication during the six-month follow-up period.

Table 2 outlines complications occurring within one month after surgery. No significant differences were noted between the groups regarding the incidence of the shallow anterior chamber, choroidal detachment, Seidel treatment with a soft contact lens, hyphema, and vitreous hemorrhage (all p > 0.2, Fisher’s exact test).

**Table 2:** Complications within one month after surgery. All data are presented as n (%). All p-values were calculated using Fisher’s exact test. TLE = trabeculectomy

Complications of the surgery	PreserFlo	TLE	P-value
Shallow anterior chamber	2 (7)	2 (7)	0.666
Choroidal detachment	5 (19)	3 (10)	0.312
Seidel treated by a soft contact lens	0	1 (3)	0.517
Hyphema	2 (7)	3 (10)	0.534
Vitreous hemorrhage	0	2 (7)	0.263

Ocular biometry changes

**Figure 2:**
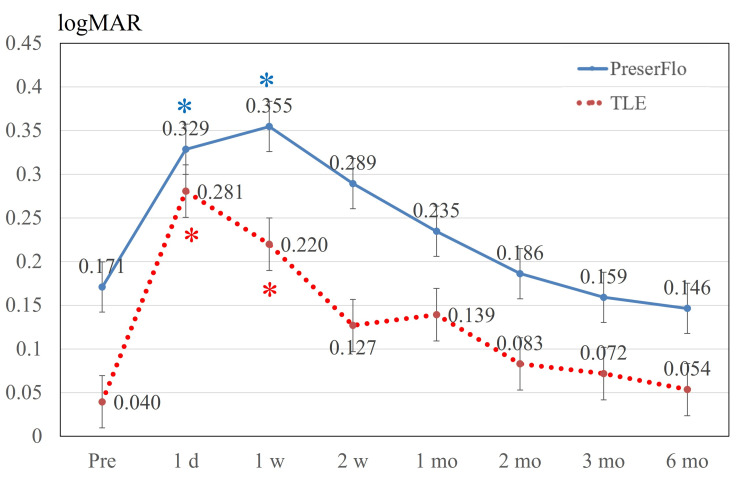
BCVA. In both groups, BCVA decreased significantly until one week after surgery, recovering to the baseline level two weeks thereafter. Significant differences compared to the baseline are indicated by blue * in the PreserFlo group and red * in the TLE group. Values are shown as mean ± standard error of the mean. BCVA = best-corrected visual acuity; TLE = trabeculectomy

**Figure 3:**
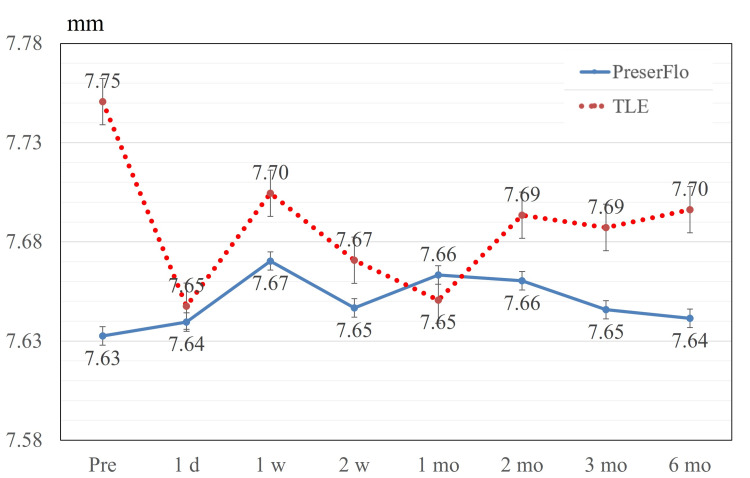
Corneal curvature. No significant difference in corneal curvature was detected in the PreserFlo and TLE groups throughout the follow-up period (p = 0.559 and 0.131, respectively). Values are shown as mean ± standard error of the mean. TLE = trabeculectomy

**Figure 4:**
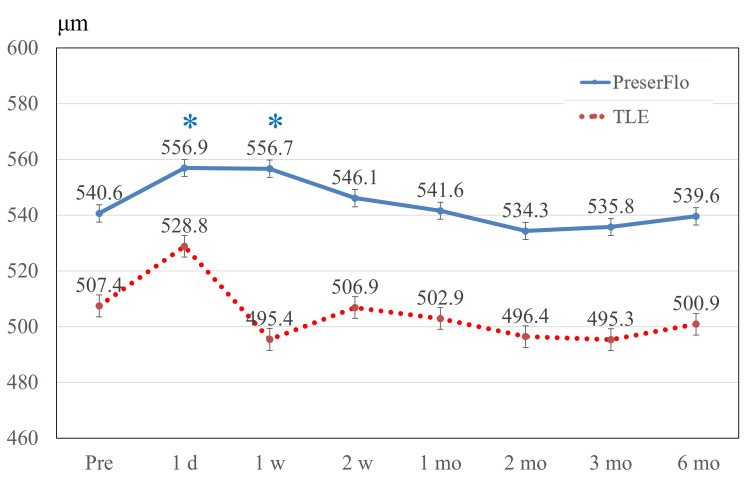
Central corneal thickness. Central corneal thickness significantly increased one day (p = 0.001) and one week (p = 0.001) after surgery in the PreserFlo group. In the TLE group, no significant difference between the baseline and other time points was observed. Significant differences compared to the baseline are indicated by blue * in the PreserFlo group. Values are shown as mean ± standard error of the mean. TLE = trabeculectomy

Figure 2 presents the alterations in BCVA after the surgical procedures. In the PreserFlo cohort, significant differences were detected over follow-up (p < 0.001 by repeated-measures ANOVA). Post hoc multiple comparisons tests revealed a significant difference in BCVA between baseline and one day after surgery (p = 0.002) and between baseline and one week after surgery (p < 0.001). In the TLE group, significant differences were detected over follow-up (p < 0.001 by repeated-measures ANOVA). Post hoc multiple comparison tests revealed a significant difference in BCVA between baseline and one day after surgery (p < 0.001) and between baseline and one week after surgery (p = 0.006). No significant variations in corneal curvature were recorded either in the PreserFlo group (p = 0.559) or the TLE group (p = 0.131) during follow-up (Figure 3). Figure 4 presents the alterations in central corneal thickness after the surgical procedure.

In the PreserFlo cohort, significant differences were detected over follow-up (p < 0.001 by repeated-measures ANOVA). Post hoc multiple comparison tests revealed a significant difference in central corneal thickness between baseline and one day after surgery (p = 0.001) and between baseline and one week after surgery (p = 0.001).

**Figure 5:**
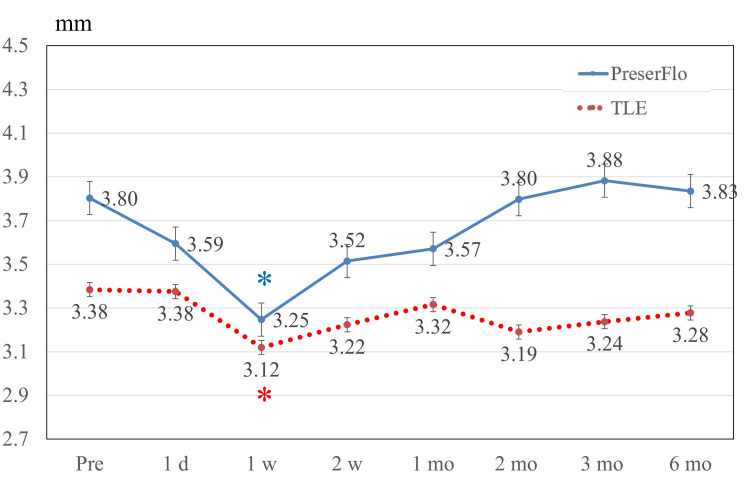
Anterior chamber depth. In both groups, anterior chamber depth decreased significantly one week after surgery (all p < 0.001, respectively), but recovered to the baseline level two weeks after surgery. Significant differences compared to the baseline are indicated by blue * in the PreserFlo group and red * in the TLE group. Values are shown as mean ± standard error of the mean. TLE = trabeculectomy

**Figure 6:**
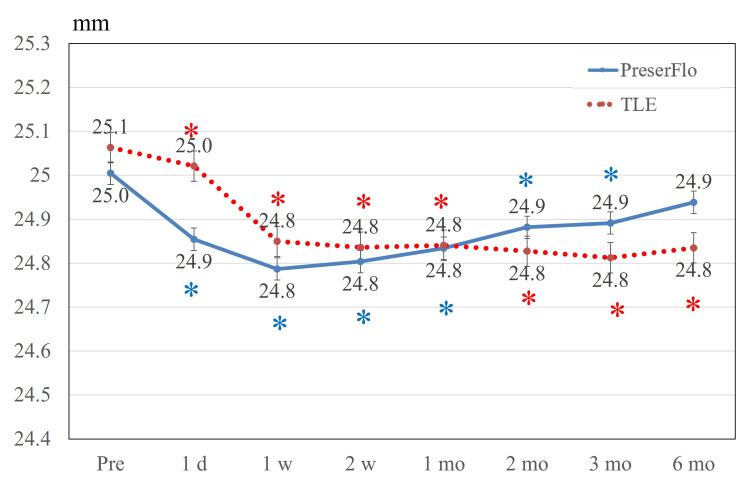
Axial length. Axial length decreased significantly until three months after surgery in the PreserFlo group and at all time points after surgery in the TLE group. Significant differences compared to the baseline are indicated by blue * in the PreserFlo group and red * in the TLE group. Values are shown as mean ± standard error of the mean. TLE = trabeculectomy

In the TLE cohort, significant differences were detected over follow-up (p < 0.001 by repeated-measures ANOVA). However, post hoc multiple comparison tests revealed no significant difference between baseline and other time points. Figure 5 delineates fluctuations in anterior chamber depth. In the PreserFlo group, statistically significant differences were observed over follow-up (p < 0.001 by repeated-measures ANOVA), and post hoc analysis identified a significant difference in anterior chamber depth between baseline and one week after surgery (p < 0.001). In TLE, there was significant difference during the follow-up period (p = 0.002 by repeated-measures ANOVA) and post hoc analysis identified a significant difference in anterior chamber depth between baseline and one week after surgery (p = 0.005). Figure 6 presents changes in axial length after surgical procedures. In the PreserFlo cohort, significant differences were detected over follow-up (p < 0.001 by repeated-measures ANOVA). Post hoc multiple comparison tests revealed a significant difference in axial length between baseline and at one day, one week, two weeks, one month, two months, and three months after surgery (all p < 0.001).

In the TLE cohort, significant differences were detected over follow-up (p < 0.001 by repeated-measures ANOVA). Post hoc multiple comparison tests revealed a significant difference in axial length between baseline and all time points after surgery (all p < 0.001).

## Discussion

This study presented detailed data on changes in ocular biometry over time after PreserFlo and TLE surgery. Few reports have described biometric changes after PreserFlo and TLE surgery. In prior reports, BCVA was unchanged from baseline to 6 [11,20] or 12 months [20,21] after PreserFlo surgery. However, Ibarz Barberá et al. reported that BCVA significantly decreased at one week after PreserFlo implantation, but it recovered at one and three months [17], which were similar to the PreserFlo cohort where it decreased significantly at one day and one week postoperatively. In prior research, BCVA was not changed versus baseline at 6 [11] and 12 months [21] after TLE. However, Sato et al. reported a significant decrease at one week, one month, and three months postoperatively [22]. In our TLE cohort, BCVA decreased one day and one week after surgery, probably because of early complications after the surgery. We believe the better BCVA results over time in our study are attributable to the experience of the surgeon and the reduction in surgically induced astigmatism achieved by full laser suture lysis.

Both PreserFlo and TLE did not significantly change corneal curvature as measured by an autorefractor keratometer during follow-up. Ibarz Barberá et al. found no change in the refractive cylinder three months after PreserFlo implantation, similar to our results. However, when analyzed using a Pentacam Scheimpflug camera, anterior surface astigmatism and total corneal astigmatism (4-mm center circle) were increased at one week and three months [17]. Gambini et al. also reported that PreserFlo implantation did not change corneal curvature at one week, as measured by a kerato-refractometer [18], coinciding with our results. For eyes subjected to TLE, a review found that 0.38-1.4 diopters with-the-rule astigmatism might be induced postoperatively [23]. Further analysis of changes in astigmatism following PreserFlo surgery is necessary. Increased central corneal thickness was observed only in the PreserFlo group after one day and one week, making this the first report to present changes over time in central corneal thickness after PreserFlo implantation. Probably, the IOP at the end of the surgery (higher than the TLE group) may be the cause of central corneal thickness.

In eyes subjected to TLE, central corneal thickness was significantly increased at one week, but the value returned to baseline at one month [24]. Our data also showed a slightly increasing, but not significant (p = 0.830 by Bonferroni correction), tendency for TLE baseline 507 µm vs. 528 µm. Another report recorded no significant changes in central corneal thickness over time in TLE [25]. PreserFlo was linked to a significant decrease in anterior chamber depth at one week, which coincided with the time of the lowest IOP of 9.9 mmHg, indicating the filtering peak of PreserFlo is one week after surgery. Previous reports revealed that anterior chamber depth did not change between baseline and three months [17] and between baseline and one day or one week [18]. In TLE, a review identified a decrease in anterior chamber depth in the early days after surgery, but the value recovered to around its preoperative level after two weeks [23]. Similarly, our data also showed a significant decrease one week postoperatively.

In terms of axial length, significant decreases up to three months in the PreserFlo (about 0.1 mm) and up to six months in the TLE (0.3 mm) were observed. One report found that axial length substantially decreased by 0.13 ± 0.23 mm from baseline to three months after PreserFlo [17]. Similarly, previous reports identified a slight axial length reduction of approximately 0.2 mm in TLE [23,25,26]. Probably due to IOP decreasing, eyeballs will shrink in both groups; however, the effect will be stronger in TLE than PreserFlo because of the longer and stronger surgical effect for IOP decreasing in TLE.

This study had several limitations. First, given that our study was not randomized, and it used real-world data, differences in patient background attributable to the selection of surgery could not be avoided. We selected TLE in patients with severe visual field disturbances. The second limitation concerns the relatively small number of patients. However, this is a detailed study of changes in ocular biometry after PreserFlo. The third limitation concerning lens status may significantly affect anterior chamber depth variations in patients undergoing glaucoma surgery, especially considering the less stable anterior chamber depth in pseudophakic eyes.

## Conclusions

The postoperative changes in ocular biometry after PreserFlo MicroShunt implantation and TLE had similar postoperative tendencies. Our results will assist surgeons in the surgical prediction of new and conventional filtering surgery.

## References

[1] (2023). World Glaucoma Association. What is glaucoma. https://wga.one/what-is-glaucoma/.

[2] de Voogd S, Ikram MK, Wolfs RC, Jansonius NM, Hofman A, de Jong PT (2005). Incidence of open-angle glaucoma in a general elderly population: the Rotterdam Study. Ophthalmology.

[3] Bengtsson B, Heijl A (2005). A long-term prospective study of risk factors for glaucomatous visual field loss in patients with ocular hypertension. J Glaucoma.

[4] Leske MC, Wu SY, Hennis A, Honkanen R, Nemesure B (2008). Risk factors for incident open-angle glaucoma: the Barbados Eye Studies. Ophthalmology.

[5] Leske MC, Heijl A, Hussein M, Bengtsson B, Hyman L, Komaroff E (2003). Factors for glaucoma progression and the effect of treatment: the early manifest glaucoma trial. Arch Ophthalmol.

[6] Kerr NM, Wang J, Barton K (2017). Minimally invasive glaucoma surgery as primary stand-alone surgery for glaucoma. Clin Exp Ophthalmol.

[7] Chan PP, Larson MD, Dickerson JE Jr (2023). Minimally invasive glaucoma surgery: latest developments and future challenges. Asia Pac J Ophthalmol (Phila).

[8] Balas M, Mathew DJ (2023). Minimally invasive glaucoma surgery: a review of the literature. Vision (Basel).

[9] Bonnar J, Azuara-Blanco A (2023). Systematic review of the method and quality of reporting of complications from studies evaluating innovative glaucoma surgical procedures. Eye (Lond).

[10] Nobl M, Freissinger S, Rudolph K, Vounotrypidis E, Kassumeh S, Priglinger S, Mackert MJ (2023). Long-term outcomes of PreserFlo MicroShunt versus XEN45 gel stent in open-angle glaucoma [in press]. Klin Monbl Augenheilkd.

[11] Wagner FM, Schuster AK, Munder A, Muehl M, Chronopoulos P, Pfeiffer N, Hoffmann EM (2022). Comparison of subconjunctival microinvasive glaucoma surgery and trabeculectomy. Acta Ophthalmol.

[12] Panarelli JF, Moster MR, Garcia-Feijoo J (2024). Ab-externo MicroShunt versus trabeculectomy in primary open-angle glaucoma: two-year results from a randomized, multicenter study. Ophthalmology.

[13] Pawiroredjo SS, Bramer WM, Pawiroredjo ND (2022). Efficacy of the PRESERFLO MicroShunt and a meta-analysis of the literature. J Clin Med.

[14] Strzalkowska A, Strzalkowski P, Hoffmann EM, Pfeiffer N, Schuster AK (2023). Outcomes of open bleb revision after PreserFlo MicroShunt failure in patients with glaucoma. J Glaucoma.

[15] Kuet ML, Azuara-Blanco A, Barton K, King AJ (2023). Will the PRESERFLO™ MicroShunt impact the future of trabeculectomy practice? A UK and Éire Glaucoma Society National Survey. Eye (Lond).

[16] Ahmed II, Sadruddin O, Panarelli JF (2023). Subconjunctival filtration in evolution: current evidence on MicroShunt implantation for treating patients with glaucoma. Eye Vis (Lond).

[17] Ibarz Barberá M, Morales-Fernandez L, Gómez de Liaño R, Tañá Rivero P, Teus MA (2021). Changes to corneal topography and biometrics after PRESERFLO Microshunt surgery for glaucoma. J Glaucoma.

[18] Gambini G, Carlà MM, Giannuzzi F (2023). Early post-operative anterior segment parameters modifications induced by PreserFlo MicroShunt in primary open-angle glaucoma. Int Ophthalmol.

[19] Negretti GS, Chan W, Pavesio C, Muqit MM (2020). Vitrectomy for endophthalmitis: 5-year study of outcomes and complications. BMJ Open Ophthalmol.

[20] Bhayani R, Martínez de la Casa JM, Figus M, Klabe K, Rabiolo A, Mercieca K (2023). Short-term safety and efficacy of Preserflo™ Microshunt in glaucoma patients: a multicentre retrospective cohort study. Eye (Lond).

[21] Jamke M, Herber R, Haase MA, Jasper CS, Pillunat LE, Pillunat KR (2023). PRESERFLO ™ MicroShunt versus trabeculectomy: 1-year results on efficacy and safety. Graefes Arch Clin Exp Ophthalmol.

[22] Sato N, Kasahara M, Kono Y, Hirasawa K, Shoji N (2023). Early postoperative visual acuity changes after trabeculectomy and factors affecting visual acuity. Graefes Arch Clin Exp Ophthalmol.

[23] Alvani A, Pakravan M, Esfandiari H, Safi S, Yaseri M, Pakravan P (2016). Ocular biometric changes after trabeculectomy. J Ophthalmic Vis Res.

[24] Simsek M, Elgin U, Uzel MM, Sen E, Yilmazbas P (2018). Stabilization time of anterior segment parameters after trabeculectomy surgery. Eye Contact Lens.

[25] Kang YS, Helong P, Sung MS, Park SW (2021). Posture-induced changes in intraocular pressure after trabeculectomy in patients with primary open-angle glaucoma. J Glaucoma.

[26] Pakravan M, Alvani A, Esfandiari H, Ghahari E, Yaseri M (2017). Post-trabeculectomy ocular biometric changes. Clin Exp Optom.

